# Supplementary material for: Content-rich biological network constructed by mining PubMed abstracts
Source: BMC Bioinformatics. 2004 Oct 8;5:147. doi: 10.1186/1471-2105-5-147 (PMC528731; doi:10.1186/1471-2105-5-147)
Supplement: Additional File 2 — The original results of the above study (non-essential files are deleted to keep the file size under the limit set by BMC bioinformatics). [file 1471-2105-5-147-S2.bz2 › chilibotAdditionalFile2/dip05/280ID2498083E1103/html/FOSB_JUNB.html]

 


 **FOSB** and **JUNB** 
  
Found 123 abstracts in PubMed, retrieved 05.  
 

 What does Google say? 
 PDF only 
| .edu only 

---

**Interactive relationship** (e.g. stimulation, inhibition, etc)

**Non-interactive relationship** (e.g. studied together, co-existance, homology, etc.)

- Phosphorylated c Jun,  **JunB** , JunD, and Fra 1, but not c Fos,  **FosB** , or ATF 2, are detected in the AP 1 DNA binding complex in cells exposed to trivalent arsenicals.  Ref: 12475910 FASEB J, 2003
- Immunoblot analyses confirmed that c Jun, JunD,  **JunB** , c Fos, Fra 1, Fra 2 and  **FosB**  immunoreactive proteins were present in whole cell extracts of all antral follicles and midluteal phase corpora lutea as well as granulosa cells isolated from different sized antral follicles.  Ref: 12606371 Biol Reprod< MedlineTA>Biol Reprod, 2003
- Among the early response genes analyzed, c myc,  **junB** , junD, c jun, c fos,  **fosB** , fra, as well as max, mad1 4, sin3, only c jun and fra 2 mRNAs were up regulated after 5D exposure.  Ref: 12648525 J Steroid Biochem Mol Biol, 2003
- We further studied the expression of  **fosB** , c jun and  **junB** , in the AP 1 complex.  Ref: 12820376 Anticancer Res< MedlineTA>Anticancer Res, 2003
- This effect was specific because palytoxin had little effect on c Jun,  **JunB** , JunD,  **FosB** , Fra 1, or Fra 2 binding or on overall levels of transcription factor binding.  Ref: 12460732 Toxicol Appl Pharmacol, 2002
